# Optimizing Cervical Cancer Screening and Triage in Low-Resource Settings

**DOI:** 10.1200/JGO.18.00192

**Published:** 2018-09-25

**Authors:** Julia S. Seay, Erin Kobetz

**Affiliations:** Julia S. Seay and Erin Kobetz, University of Miami School of Medicine, Miami, FL

Widespread adoption of screening has led to dramatic decreases in the global burden of cervical cancer.^1^ However, this decline has not been experienced equitably by all women worldwide. Those living in low- and middle-income countries and/or low-resource settings remain at increased risk for developing and dying as a result of cervical cancer, largely a result of a lack of access to routine screening and follow-up monitoring and treatment of dysplastic lesions.^2,3^ The Papanicolaou test/cytology, which remains the gold standard for cervical cancer prevention and early detection, requires extensive resources, including highly trained physicians and complex laboratory equipment.^2^ Places without such infrastructure have historically been vulnerable to cervical cancer disparity.

Several previous studies have examined the relative feasibility, acceptability, and efficacy of various primary screening strategies, including Papanicolaou test, human papillomavirus (HPV) self-sampling, and visual inspection with acetic acid (VIA), in improving cervical cancer screening uptake and dissemination within resource-limited settings.^4-6^ Many of these have found that point-of-care HPV testing, especially when paired with self-sampling, is both highly sensitive and cost effective for primary cervical cancer screening. However, few previous studies have examined optimal methods for the triage of women with abnormal primary screenings. The article by Poli et al^7^ fills that gap. It presents an innovative study comparing VIA, cytology, and colposcopy for triage among a large sample of underserved women who screened positive by point-of-care HPV testing in Hyderabad, India. The primary aim of this inquiry was to determine the optimal triage strategy within this and other comparable, low-resource settings.

The researchers considered a multitude of factors when evaluating each triage strategy, including test sensitivity and logistic barriers (amount of time taken, need for specialized labor, requirement to return to the clinic for results delivery and treatment) to implementation. Although they found that VIA was indeed less sensitive than cytology for identifying cervical dysplasia, Poli et al^7^ concluded that VIA was the optimal triage technique for the study setting, given that primary screening with point-of-care HPV testing, VIA triage, and treatment could all be conducted within the same clinic visit. Moreover, point-of-care HPV testing with VIA triage was identified as the most cost effective among each screening algorithm considered.

Study findings highlight the importance of considering contextual factors in determining the optimal screening algorithms in resource-limited settings. Although cytology performed substantially better than VIA in terms of sensitivity, both the cost and feasibility of cytology for triage of women with HPV-positive disease were prohibitive within this low-resource context. Ultimately, although point-of-care HPV testing is likely to usurp VIA as the best practice for primary screening in many low-resource settings, the current study demonstrates how VIA can be used to effectively triage women for colposcopy and treatment when access to cytology is challenged.

Another notable aspect of the study was the use of community health workers and mobile screening strategies to promote the uptake of cervical cancer screening among the local population. HPV self-sampling has been shown to have high acceptability across a variety of marginalized populations worldwide, and when delivered by culturally competent health workers, has been shown to circumvent structural and cultural barriers to cervical cancer prevention, such as beliefs regarding modesty and discomfort with the speculum examination. Moreover, the establishment of mobile screening protocols will likely increase the effectiveness and reach of the point-of-care HPV testing/VIA triage approach. Notably, neither of these screenings require a clinic setting to be successfully conducted.

Ultimately, these findings lend themselves to several areas of future inquiry. The large-scale effectiveness of the point-of-care HPV testing/VIA triage method has yet to be established. Moreover, this triage method may also be advantageous within other resource-limited settings. For example, although VIA has been successfully used in rural Haiti as part of a screen-and-treat approach, the addition of point-of-care HPV testing as a primary screening strategy has not yet been examined. It is likely that this screening algorithm will have a substantial impact on improving the sensitivity and effectiveness of low-resource screening programs worldwide. Finally, although the current point-of-care HPV test, careHPV, has been shown to be effective in a variety of contexts, the test requires 2.5 hours to be completed. This is a vast improvement over traditional HPV testing, although it may still prohibit same-day triage and treatment in certain contexts. Thus, future work is needed to optimize point-of-care testing and reduce the amount of time necessary to receive test results.
